# Molecular Mechanism and Pathways of Spontaneous Preterm Birth in Different Gestational Tissues: A Systematic Review of Transcriptome Studies

**DOI:** 10.3390/ijms27136006

**Published:** 2026-07-04

**Authors:** Yue Wang, Hillary Hiu Yu Leung, Annie Shuk Yi Hui, Lo Wong, Tak Yeung Leung

**Affiliations:** Department of Obstetrics and Gynaecology, Faculty of Medicine, The Chinese University of Hong Kong, Shatin, Hong Kong, China

**Keywords:** spontaneous preterm birth, spontaneous preterm labor, preterm prelabor rupture of membranes, transcriptomics, differentially expressed genes, inflammation, extracellular matrix remodeling

## Abstract

This systematic review assessed transcriptomic evidence on the molecular mechanisms underlying spontaneous preterm birth (sPTB). Major electronic databases were searched from inception to October 2025. Eligible studies examined RNA transcriptomic profiles from maternal pregnancy-related tissues or biofluids in spontaneous preterm labor (sPTL) or preterm prelabor rupture of membranes (PPROM), while indicated or iatrogenic preterm births were excluded. Two reviewers independently screened studies, extracted differentially expressed genes (DEGs), and assessed study quality. DEGs were summarized by tissue type, and recurrent concordant genes were analyzed using Gene Ontology, Reactome, and Kyoto Encyclopedia of Genes and Genomes enrichment analyses, with false discovery rate < 0.05 considered significant. Twenty studies were included. Transcriptomic data were derived from placental villi, maternal peripheral blood, decidua, fetal membranes, myometrium, amniotic fluid, and vaginal secretions. Placental villi findings suggested proliferative-metabolic reprogramming and impaired maternal–fetal immune–structural homeostasis, whereas maternal blood profiles reflected systemic immune–inflammatory activation and dysregulated lipid-metabolic pathways. sPTL and PPROM showed potentially distinct signatures involving extracellular matrix disruption, collagen remodeling, matrix degradation, and myeloid/neutrophil-associated inflammation. Transcriptomic profiling may support non-invasive sPTB risk assessment, but standardized, phenotype-specific longitudinal studies are needed to confirm predictive value and clinical utility.

## 1. Introduction

Preterm birth (PTB), defined as delivery before 37 completed weeks of gestation, affects 10–11% of pregnancies worldwide and remains a leading cause of neonatal and under-five mortality despite advances in perinatal care [1,2,3]. While approximately one-third of PTBs are medically indicated and iatrogenic, the remaining two-thirds are spontaneous (sPTB), occurring either after spontaneous onset of preterm labor (sPTL) or in the context of preterm prelabor rupture of membranes (PPROM)—the most common identifiable cause of sPTB [3,4,5]. Both sPTL and PPROM are heterogeneous syndromes driven by interacting pathways, including dysregulated maternal–fetal immune tolerance, infection and sterile inflammation, oxidative stress, extracellular matrix (ECM) remodeling, and premature senescence across the uterus, cervix, fetal membranes, and placenta [6,7,8,9].

To discover the complex pathophysiology and to predict the occurrence of PTB, high-throughput transcriptomic technologies have been the crucial tool by enabling unbiased, genome-wide characterization of differential gene expression in gestational tissues and biofluids, including placental villis, chorioamniotic membranes, decidual, cervical and myometrial tissues, and maternal blood [10,11,12,13,14,15,16,17,18,19]. However, previous transcriptomic studies of PTB have been limited by both study design and technical constraints. A 2015 systematic review by Eidem et al., which included a substantial proportion of indicated PTB cases and relied predominantly on microarray-based datasets, concluded that reproducible differentially expressed genes (DEGs) specific to sPTB were difficult to identify across studies [20]. Although Vora et al. (2018) reported a consistent immune signature characterized by upregulated innate immunity and downregulated adaptive immunity through reanalysis of three public datasets, their findings were largely restricted to maternal blood and did not fully resolve tissue-specific versus systemic effects [21]. Similarly, the 2020 systematic review by Brummaier et al. focused on maternal blood transcriptomics, again included indicated PTB cases, and lacked cross-tissue comparisons [22]. In 2021, Tarca et al. attempted to analyze PTB using multi-omics data, including transcriptomics; however, their focus was on microRNAs rather than mRNA [15]. Collectively, these limitations underscore the need for integrative analyses that specifically address sPTB and systematically compare immune-related transcriptomic signatures across maternal and fetal compartments.

Integrated genome–transcriptome analyses and cross-compartment studies are increasingly uncovering molecular pathways underlying sPTB and beginning to define biologically meaningful endotypes. Focuses on primary bulk mRNA transcriptomic studies, including microarray and bulk RNA-seq studies, this systematic review aims at summarizing the reported transcriptomic alterations across tissues and biofluids in sPTB, and discussing the translational potential as well as the current limitations of transcriptome-based approaches for early prediction, risk stratification, and mechanistic insight.

## 2. Materials and Methods

### 2.1. Search Strategy

The search strategy was developed using the Population, Phenomenon of Interest, and Context (PICo) framework to ensure comprehensive identification of relevant published research. For this systematic review, we conducted searches in Web of Science, PubMed, Embase, Scopus and GEO databases from database inception to October 2025. The search was subsequently updated and monitored until May 2026 during manuscript preparation; however, no additional eligible studies were identified for inclusion in the analysis. The review protocol has been registered with the International Prospective Registry of Systematic Reviews (PROSPERO), with the registration number CRD420251245728.

The search terms were grouped into four concept blocks: preterm birth-related terms, RNA sequencing/transcriptomic profiling-related terms, differential expression or pathway analysis-related terms, and tissue/sample source-related terms. Terms within each block were combined using the Boolean operator “OR”. The concept blocks were then combined using “AND” in prespecified two-concept and three-concept combinations to maximize search sensitivity. All records retrieved from the separate searches were exported, merged, and deduplicated before title and abstract screening.

Search results were restricted to studies published in English. In addition, the reference lists of all included articles were manually screened to identify any potentially relevant publications not captured by the primary database search. All eligible records were imported, organized, and managed using EndNote software (version 21.5).

For GEO, the same concept-block search strategy was applied where applicable. Records were screened according to organism, sample source, study design, and availability of extractable gene-level transcriptomic results. Only human studies relevant to sPTB and bulk mRNA transcriptomic profiling were considered eligible.

### 2.2. Study Selection and Data Extraction

Two reviewers independently performed the literature screening and data extraction for all eligible studies. Disagreements were resolved through consultation with a third reviewer, followed by discussion and consensus among all authors. To assess study quality, we developed a customized appraisal scale (Table S1) based on the Strengthening the Reporting of Observational Studies in Epidemiology (STROBE) guidelines, the Newcastle–Ottawa Scale (NOS), and the Minimum Information About a Microarray Experiment/Minimum Information About a high-throughput Nucleotide Sequencing Experiment (MIAME/MINSEQE) guidelines, with additional criteria tailored to PTB transcriptomic research. All included studies were evaluated using this scale. The following information was extracted when available: (a) study-level characteristics, including authors, year of publication, sample size, gestational age at delivery, mode of delivery, and the type, anatomical source, and collection methods of the biological specimens; (b) methodological details, particularly the platforms and technologies used for gene-expression profiling; and (c) statistical outputs from differential-expression analyses, such as lists of significantly altered genes, direction of change, FC estimates, and corresponding raw and adjusted *p*-values.

### 2.3. Subgroup Allocation

To align with the objectives and analytical focus of the included studies, a subgroup classification scheme was established. Each article was evaluated to determine whether its transcriptomic analysis (a) elucidated factors implicated in sPTB across different pregnancy-related tissues; (b) supported the prediction of PTB or PPROM; or (c) included experimental validation of the differential transcriptome. Studies could be assigned to more than one subgroup as appropriate.

### 2.4. Data Synthesis

We initially compiled all reported DEGs according to each study’s predefined significance criteria when comparing cases with and without sPTB. Subsequently, we identified and extracted genes that were consistently reported in at least two studies examining the same tissue type. For genes that exhibited concordant directional changes in RNA expression across multiple studies of the same tissue, functional enrichment analyses were performed. GO, Reactome pathway enrichment (an expert-curated repository that organizes genes into biologically meaningful pathways), and KEGG enrichment analyses were conducted using the Database for Annotation, Visualization and Integrated Discovery (DAVID). To further assess the robustness of pathway enrichment, validation analyses were carried out for DEGs using R packages (version 0.84.1). Multiple-testing correction was applied using the false discovery rate (FDR), and only results with an FDR < 0.05 were considered statistically significant.

## 3. Results

### 3.1. Selection of Studies

**Figure 1:**
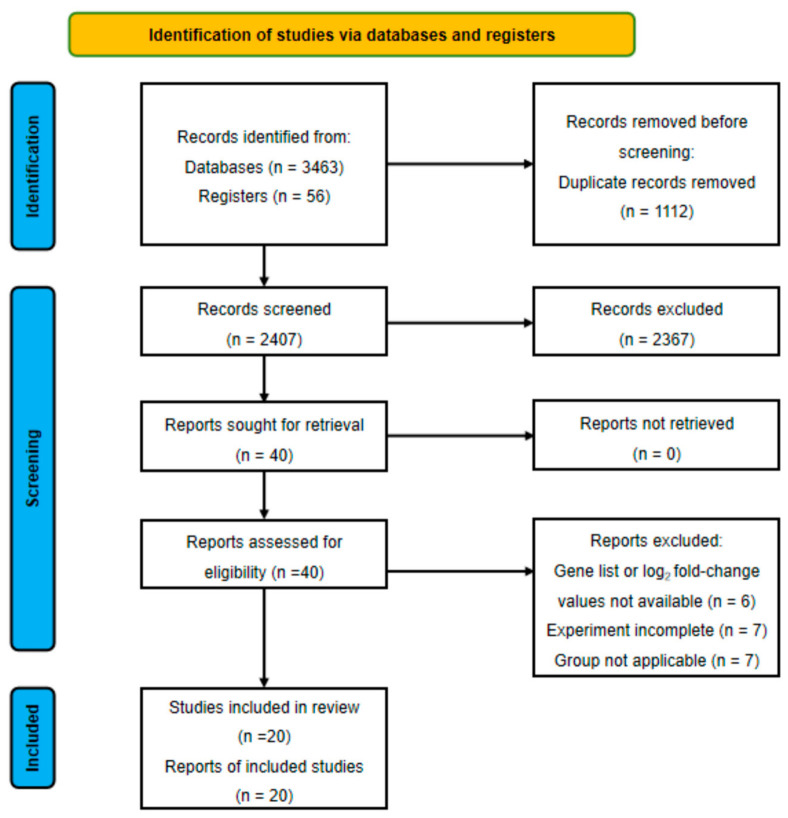
Study selection flow diagram according to PRISMA 2020 [58].

The database search was completed in October 2025. After initial retrieval and removal of duplicate records, 2407 citations were screened. Of these, 2367 were excluded based on title and abstract review. A total of 40 full-text articles were subsequently assessed for eligibility, and 20 of them were further excluded for the following reasons: five studies did not provide a list of DEGs and [23,24,25,26,27] one article did not list the log_2_ fold-change (FC) values of the DEGs [28]. Seven studies did not meet experimental requirements. Among them, three studies performed only quantitative polymerase chain reaction (qPCR) validation experiments without conducting exploratory sequencing in preterm groups [24,29,30]; two articles focused on single-cell or spatial transcriptomes [31,32]; and two studies focused on analyzing others’ data for model training and validation [14,33]. In addition, seven articles were excluded based on their study focus or design. Of those, four articles focused on medically indicated PTB [34,35,36,37]; two articles primarily analyzed the maturity of preterm fetuses through amniotic fluid transcriptome [38,39]; and one article compared the transcriptional differences between resting and active states of the myometrium rather than directly comparing PTB and term birth (TB) [40]. Finally, 20 studies met the inclusion criteria (Figure 1 and Table 1).

**Table 1:** The basic characteristics of the 20 included studies on differentially expressed genes in spontaneous preterm birth, listed according to the grouping and in chronological order.

Authors, Country, Year	Groups	No. of Subjects, *n*	GA at Delivery, wks	Mode of Delivery, n	Sample Types	Collection Time	No. of DEGs		Profiling Methods	Definition of DEGs	Validation Methods
							Up	Down			
Group A1											
Chim et al., HK, China (2012) [41]	sPTB	10	31.2 (27.0–32.6)	SVD (10)	Placental villi	Immediately upon delivery	240	186	Microarray	P_adj_ < 0.05 & FC ≥ 2.9	qRT–PCR
	TB	20	38.8 (38.4–39.3)	SVD (10) CS (10)							
Brockway et al., USA (2019) [42]	sPTB	12	33 (30–36)	SVD (7) CS (5)	Placental villi	Within 60 min of delivery	185	5	RNA-seq	P_adj_ < 0.05 & |log_2_FC| ≥ 1	qRT–PCR & IHC
	TB	11	39 (38–42)	SVD (5) CS (6)							
Lien et al., USA (2021) [43]	sPTB–M	8	29.4 (25.1–33.7)	SVD (9) CS (6)	Placental villi	At the time of delivery	327	377	RNA-seq	P_adj_ ≤ 0.05	Proteomics
	TB–M	7	39.7 (39–40.4)								
	sPTB–F	8	32.1 (28.1–36.1)	SVD (13) CS (3)			28	38			
	TB–F	8	39.5 (38.8–40.2)								
Couture et al., Canada (2023) [44] ^1^	sPTB	38	32.4 (26.1–36.7)	SVD (18) CS (20)	Placental villi and fetal membrane	Shortly after delivery	102	229	RNA-seq	P_adj_ < 0.05 & |log_2_FC| ≥ 1	Flow cytometry, Histologic analysis
	TB	41	39.8 (38–41.4)	SVD (16) CS (25)							
Akram et al., UK (2022) [45]	sPTB	20	33.5 (24–36.6)	SVD (17) CS (3)	Placental villi	Within 3 h of delivery	337	564	RNA-seq	P_adj_ < 0.05 & |log_2_FC| ≥ 1	qRT–PCR & ELISA
	TB	20	39.6 (37.4–41.1)	SVD (19) CS (1)							
Paquett et al., USA (2023) [13]	Early sPTB	12	<34	SVD (34) CS (14)	Placental villi	Within 15 min of delivery	PTB vs. TB		RNA-seq	P_adj_ < 0.05	NA
							295	666			
	Late sPTB	36	34–<37				Early PTB vs. TB				
	Early TB	339	37–<39	SVD (316) CS (214)			464	776			
	Full TB	181	39–<41				Late PTB vs. TB				
	Late TB	20	41–<42				6	16			
Group A2											
Rinaldi et al., UK (2017) [46]	sPTB-L	10	30.4 (24.0–34.6)	SVD (10) CS (7)	Decidual lymphocytes	Following delivery	TB-L vs. TB-NL		Microarray	*p* < 0.05 & FC ≥ 1.2	qPCR + WB
	PTB-NL	7	32.4 (29.0–34.4)				56	48			
							sPTB-L vs. TB-L				
	TB-L	8	40.1 (38.2–41.1)	SVD (8) CS (11)			32	80			
							sPTB–L vs. PTB–NL				
	TB-NL	11	39.3 (39.0–41.0)				96	33			
Pereyra et al., Uruguay (2019) [10]	sPTB	9	30.7 (29.8–31.6)	SVD (9)	Fetal membranes (1 cm^2^)	Within 30 min post delivery	252	18	RNA-seq	P_adj_ < 0.05 & |log_2_FC| > 2	qRT–PCR
	TB	15	39.3 (39–39.6)	SVD (15)							
Arrowsmith et al., UK (2020) [47]	sPTL-S	6	35.9 (34.4–37.1)	SVD (0) CS (12)	Myometrium	Following delivery	sPTB–S vs. TB–T		RNA-seq	P_adj_ < 0.05	NA
	sPTL-T	6	35.6 (33.4–37.4)								
	TB-S	6	38.3 (38–38.6)	SVD (0) CS (12)			24	75			
	TB-T	6	38 (37.7–38.3)								
Bhatti et al., USA (2021) [48]	Delivered within 24 h from AM	10	32.8 (27.9–33.9)	SVD (8) CS (2)	Amniotic Fluid	After an episode of preterm labor	1508	877	Microarray	P_adj_ < 0.1 & FC ≥ 1.25	NA
	Delivered after 24 h from AM	28	34.8 (31–38.8)	SVD (25) CS (3)							
Wikstrom et al., Sweden (2022) [19]	sPTB	48	<37^+0^	SVD (42) CS (6)	Vaginal fluid specimens	18–20^+6^ weeks	15	2	RNA-seq	P_adj_ < 0.05	NA
	TB	96	39^+0^–40^+6^	SVD (93) CS (3)							
Group B1											
Heng et al., Canada (2014) [49]	sPTL in 48 h	48	31.8 (28.5–35.1)	NA	Whole maternal blood	At point of hospital admission	256	213	Microarray	P_adj_ < 0.05	qRT–PCR
	sPTL 48 h–7 d	12	29.5 (26.4–32.6)								
	sPTL 7 d–37 w	15	34.8 (32.7–36.9)								
	TB	79	39.0 (37.8–40.2)								
Paquette et al., Canada (2018) [50]	sPTL	20	23–34	SVD (17) CS (3)	Whole maternal blood cells & peripheral monocytes	At the point of hospital admission	40	3	RNA-seq	P_adj_ < 0.05 & |log_2_FC| > 1	qRT–PCR
	TB	30	>37	SVD (12) CS (17) NA (1)							
Yoo et al., Korea (2021) [51]	sPTB	5	31.6 (30.2–33)	NA	Whole maternal blood	When admitted to the hospital	273	660	RNA-seq	P_adj_ < 0.05 & FC > 1.5	qRT–PCR
	TB	5	39.3 (39–39.6)								
Group B2											
Heng et al., Canada (2016) [52]	sPTL	51	33.6 (31–36.2)	NA	Whole maternal blood	T1: 17–23 wks	T1: no DEG observed		Microarray	P_adj_ < 0.05	qRT–PCR
	TB	114	39.2 (38–40.4)			T2: 27–33 wks	21	5			
Weiner et al., USA (2021) [53]	sPTB	5	≤32	NA	Maternal blood plasma	From 18.3 ± 1.4 wks to delivery (per 2 wks)	61	235	Microarray	P_adj_ < 0.05 & FC ≥ 1.5	qRT–PCR
	TB	5	>37								
Gupta et al., UK (2022) [54]	sPTB	53	≤34^+0^	NA	Whole maternal blood	T1: 16 wks T2: 20 wks	T1 sPTB vs. HTERM		Microarray	P_adj_ < 0.05 & |log_2_FC| ≥ 1.5	NA
	HTERM	126	≥37^+0^				49	98			
	LTERM	188	≥39^+0^				Other time point and subgroups have no DEGs				
Camunas et al., UK (2022) [55]	Early sPTB	46	28.1 ± 6.0	NA	Maternal blood plasma	18.9 ± 1.9 wks	Early sPTB vs. TB		RNA-seq	|log_2_FC| > 1	NA
	TB	194	39.6 ± 1.3			20.0 ± 1.7 wks	10	15			
	Very early sPTB	14	<25 weeks			12–24 wks (no exact time point)	Very early sPTB vs TB				
	Control	193	≥25 weeks				12	26			
Group C											
Markieva et al., UK (2017) [56]	PPROM	5	<37^+0^	SVD (10) CS (1)	Cervical biopsies	Within 30 min after VD/CS	PPROM vs. sPTL		Microarray	*p* < 0.05 & FC = any	qRT–PCR
	sPTL	6					30	9			
	TB–L	12	NA	SVD (16) CS (1)			sPTL vs. TB				
	TB–NL	5					37	16			
Underhill et al., USA (2024) [57]	PPROM	4	Matched	Matched	Amnion & Chorion tissue	Within 15 min of birth	Amnion tissue		RNA-seq	*p* < 0.05 & FC ≥ 2	qRT–PCR
							991	475			
	sPTL	4					Chorion tissue				
							371	113			

^1^ Only listed the top 10 upregulated and downregulated genes. Notes: GA, gestational age; wks, weeks; DEGs, differentially expressed genes; CS, cesarean section; SVD, spontaneous vaginal delivery; FC, fold change; qRT–PCR, real-time quantitative reverse transcription polymerase chain reaction; RNA-seq, RNA sequencing; IHC, immunohistochemistry; –M, the fetus is male; –F, the fetus is female; ELISA, enzyme-linked immunosorbent assay; NA, not available; –L, spontaneous labor onset; –NL, no labor onset; WB, Western blotting; –S, singleton; –T, twin; AM, amniocentesis; HTERM, high-risk term birth; LTERM, low-risk term birth.

### 3.2. Characteristics of the Selected Studies

Among the 20 included studies, 8 were classified as high-quality and 12 as moderate-quality, while no study was considered low-quality (Table S1). They were published between 2012 and 2024 (Table 1). Sixteen of them were prospective in nature while the remaining four were retrospective. Gene expression was evaluated across multiple tissues, with a diverse set of sample sources. Most of them included placenta villi and maternal peripheral blood, with additional evidence derived from decidua, fetal membranes, myometrium, amniotic fluid, and vaginal secretions. The sample sizes varied substantially across studies, ranging from 4 to 540 participants per group.

During the study selection process, we rigorously excluded PTB attributable to maternal or fetal complications, including pre-eclampsia, gestational hypertension and known fetal genetic abnormalities. Consequently, the studies included in our analysis primarily focused on sPTB and PTB associated with PPROM. However, there were subtle differences in group settings across studies. Brockway’s study included cases later confirmed to be infection-related PTB based on histological chorioamnionitis [42]. Lien et al. further stratified PTBs and TBs by fetal sex [43]. The study by Rinaldi et al. stratified both PTBs and TBs according to the presence or absence of spontaneous labor, while the underlying causes of PTB in the non-laboring preterm group were not explicitly described [46]. Arrowsmith et al. conducted separate analyses for singleton and twin pregnancies [47]. Notably, all the research subjects in the studies included in our analysis were singletons. The study by Bhatti et al., which focused on the amniotic fluid transcriptome, included only preterm cases and compared transcriptional profiles between those who developed labor within 24 h after amniocentesis and those who did not [48]. Our analysis mainly focused on transcriptomic differences between sPTB and TB. Two studies further incorporated subgroup analyses of both sPTL and PPROM, providing insights into the shared and distinct transcriptomic features underlying the two major forms of sPTB [56,57].

All studies included in the analysis reported gestational age at delivery, which was primarily determined using last menstrual period and early-pregnancy ultrasonography. Most studies provided the median gestational age together with the corresponding observed range. Although a few studies did not report gestational age for each individual case, all provided relevant gestational-age intervals for the respective study groups.

For sample collection, all delivery-related tissues, such as placenta and fetal membranes, were obtained as soon as possible after delivery. The mode of delivery (vaginal delivery or cesarean section (CS)) was documented accordingly, but indications for CS were not specified in the original articles. For studies involving maternal blood samples, three studies collected blood at admission or prior to delivery [49,50,51], whereas four studies obtained maternal peripheral blood during the second or third trimester [52,53,54,55].

### 3.3. Technical and Methodological Aspects

The methodological characteristics of the included studies are summarized in Table 1, which details the number of up- and downregulated DEGs, the sequencing or profiling platforms used, and the criteria applied to define differential expression.

Array-based technologies (e.g., microarrays) were used in eight studies, whereas RNA sequencing (RNA-seq) was employed in twelve studies. Two studies analyzing maternal blood plasma explicitly reported the use of EDTA (ethylenediaminetetraacetic acid) tubes for sample collection, consistent with methodologies targeting cell-free RNA (cfRNA). One study reported only the top ten most significantly altered genes, while all remaining studies provided the lists of DEGs and log_2_FC value [44]. DEGs were most commonly defined using statistical significance thresholds based on *p*-values and FC cutoffs. Twelve studies performed validation of selected genes using nucleic acid amplification-based methods such as qPCR, while a smaller number of studies additionally conducted immunohistochemistry, enzyme-linked immunosorbent assay (ELISA), or Western blot assays for validation.

### 3.4. Subgroup Classification

To investigate the characteristics of gene-expression profiles, the included studies were stratified into subgroups based on PTB etiology, sample origin, and timing of sample collection. This grouping strategy was used to improve the interpretability of heterogeneous transcriptomic studies across different biological compartments. Studies in Group A focused on comparing transcriptomic differences between sPTB and TB using pregnancy-related tissue samples, without further specifying the presence or absence of rupture of membrane (ROM) prior to delivery (11 studies). Based on sample source, Group A was further subdivided into Group A1 (placental villi, six studies) and Group A2 (other pregnancy-related tissues, five studies). Group B also focused on the comparison between sPTB and TB but using maternal blood as the sample source (seven studies). Group B was further subdivided into two subgroups according to the timing of blood sampling: Group B1: during onset of labor (three studies); Group B2: before onset of labor (four studies). Group C comprised two studies that specifically compared transcriptomic differences between PPROM and sPTL without membrane rupture. Due to the diversity of sample sources and limited number of studies, Group C was not further subdivided.

#### 3.4.1. Group A and Group B—Pathway Activation in sPTB Versus TB

##### Group A1—Placenta Villi

Six mRNA-based studies examined placental villi gene expressions between sPTB and TB. Chim et al. grouped all CS subjects into a single category [41]. In contrast, the other five studies did not differentiate the mode of delivery within each subgroup. However, they did not record the specific indications for CS and only verified the comparability of delivery modes between the TB and PTB groups. Four genes—*IL1RL1*, *NFASC*, *RRM2*, and *VEGFA*—showed consistently increased expression in three of the six studies with fold changes ranging from 1.41 (*RRM2*) to 15.50 (*IL1RL1*) (Table 2). These four genes were all observed in the five studies other than the study by Couture et al. [44]. This is likely because Couture et al. reported only the top ten most significantly altered genes rather than providing a complete list of genes studied. An additional 79 genes were reported as upregulated when comparing gene expressions in preterm placentas to those in term controls in two of the six studies (Table S2). A total of 65 genes were reported to be downregulated in two out of the six studies, with FC ranging from 0.07 (*LEP*) to 0.95 (*ARF1*) (Table 3). However, no downregulated gene was reported consistently in three or more studies. Notably, the study by Brockway et al. [42] identified only five DEGs, none of which overlapped with those reported in other studies.

##### Group A2—Other Pregnancy-Related Tissues

The sample sources of the five studies in this heterogeneous group included decidual lymphocytes, fetal membranes, myometrium, amniotic fluid, and vaginal fluid specimens. Among them, the heterogeneity of these studies is readily apparent. Rinaldi et al. [46] performed subgroup analysis based on the presence or absence of spontaneous labor, while Arrowsmith et al. [47] stratified cases by singleton versus twin gestation—the only study to include twin pregnancies. In the study by Bhatti et al. [48], both groups consisted only of PTB cases. Wikstrom et al. [19] collected vaginal secretions during the second trimester but identified only a dozen DEGs. Due to heterogeneity, the consistency of DEGs was poor. Consequently, no subsequent pathway enrichment or exploratory analyses were conducted. Across the four transcriptomic studies, a consistent biological theme emerged: sPTB was repeatedly associated with activation of inflammatory and immune-related pathways rather than a single tissue-specific gene signature. In decidual and amniotic fluid transcriptomes, sPTB was characterized by broad changes in cytokine-, chemokine-, and leukocyte-related genes, whereas the myometrial study also identified enrichment of inflammation, immune response, cytokine–cytokine receptor interaction, and chemokine signaling pathways. The midgestational vaginal-fluid study further supported this pattern by showing increased expression of host transcripts related to inflammation, oxidative stress, extracellular-matrix regulation, and mucosal barrier disruption.

##### Group B—Maternal Peripheral Blood

Group B1: Among the seven studies investigating sPTB that analyzed maternal peripheral blood, three studies collected samples at the time of hospital admission when labor was imminent or already underway [49,50,51]. There were ten upregulated DEGs (*CST7*, *CYSTM1*, *EREG*, *FAM20A*, *G0S2*, *GPR84*, *IL1B*, *PER1*, *SERPINB2*, *SOCS3*) with an FC ranging from 1.31 (*CST7*) to 11.96 (*FAM20A*), while only *ABHD3* was downregulated in two of the three studies (Table 4).

Group B2: The other four studies collected samples during the second and/or early third trimester [52,53,54,55]. Only upregulation of *CAPN13* and downregulation of *LSM10* were consistently identified in two of the four studies (Table 4).

When both Groups B1 and B2 were reviewed together, two more upregulated genes (*ATN1* and *SQLE,* with FC ranging from 1,51 (*SQLE*) to 3.20 (*ATN1*)) and ten more downregulated DEGs (*AATK*, *APBB3*, *ARL11*, *CARD8*, *EBAG9*, *GPR141*, *GTF2IP4*, *LIN7A*, *RIPK3* and *S1PR4*) were consistently identified in two out of the seven studies, with FC ranging from 0.06 (*CARD8*) to 0.85 (*GTF2IP4*) (Table 4).

All FC values were extracted from the original studies and should be interpreted descriptively, as they were generated using heterogeneous platforms, normalization methods, statistical pipelines, and significance thresholds.

#### 3.4.2. Analysis of Gene Ontology (GO) Enrichment, Kyoto Encyclopedia of Genes and Genomes (KEGG) Pathways, KEGG Chord Plots and Reactome Pathways

**Figure 2:**
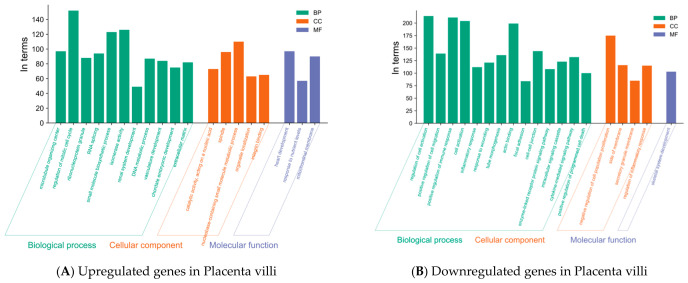

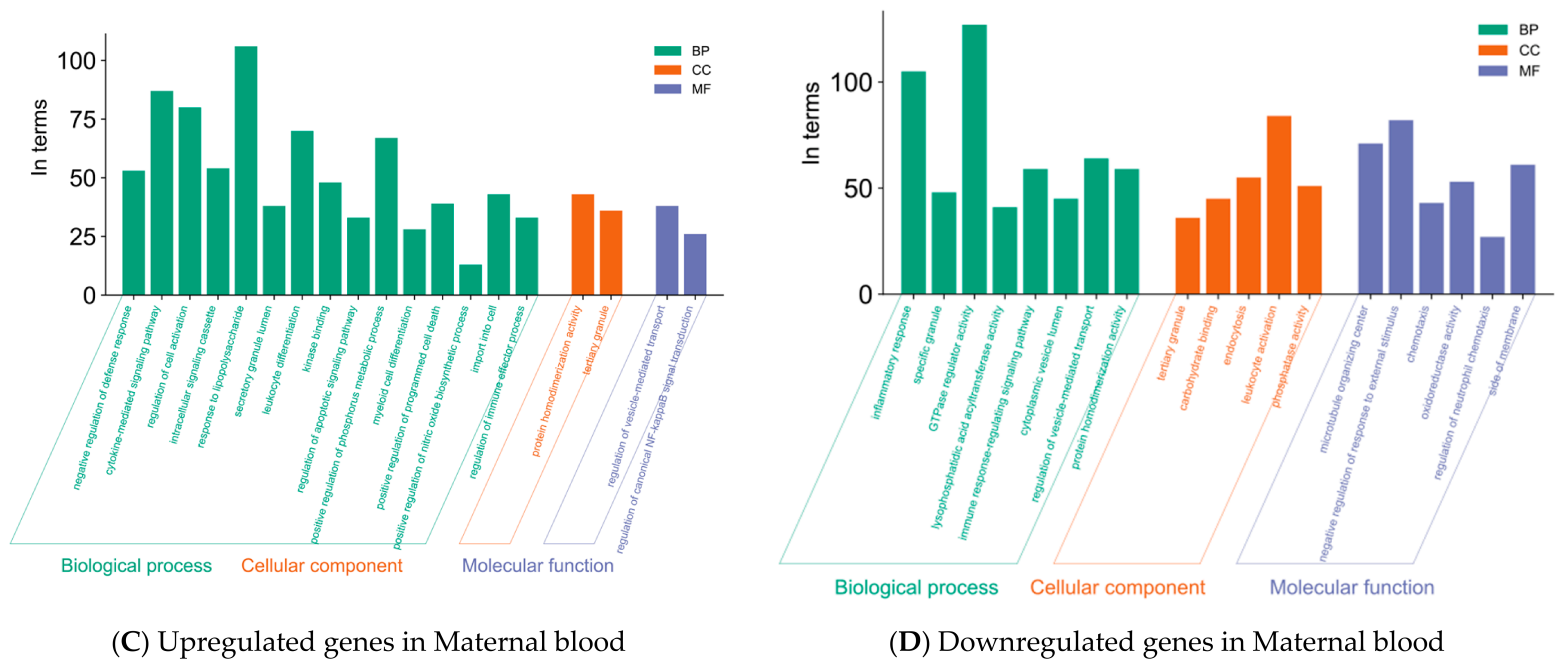
Gene Ontology (GO) enrichment analysis of genes that were identified to be differentially expressed in placental tissues and maternal blood from subjects with spontaneous preterm labor. (**A**) Up-regulated genes in placental villi; (**B**) down-regulated genes in placental villi; (**C**) up-regulated genes in maternal blood; (**D**) down-regulated genes in maternal blood.

GO enrichment analysis was performed to characterize the functional features of the DEGs across the three GO categories: biological process (BP), cellular component (CC), and molecular function (MF) (Figure 2). For each tissue group, enrichment analyses were conducted using all pooled DEGs stratified by expression direction, rather than only recurrent DEGs. In placental villous tissues from Group A1, upregulated DEGs were mainly enriched in cell cycle regulation, RNA splicing, DNA metabolism, mitochondrial function, nutrient response, and developmental processes, indicating enhanced proliferative and metabolic activity. In contrast, downregulated DEGs were primarily associated with immune activation, inflammatory response, cell migration, cell adhesion, and tissue remodeling, suggesting impaired immune homeostasis and maternal–fetal interface remodeling. These findings suggest that sPTB placentas may exhibit transcriptomic alterations related to proliferative/metabolic adaptation and defective local immune regulation; however, this interpretation should be considered exploratory given the heterogeneity of the included studies and the limited cross-study reproducibility of DEGs.

In maternal peripheral blood from Group B, GO enrichment analysis showed that the upregulated DEGs were predominantly enriched in cytokine-mediated signaling, response to lipopolysaccharide-related pathways, regulation of cell activation, leukocyte and myeloid cell differentiation, and apoptosis- and nitric oxide-related processes, indicating enhanced activation of inflammatory and innate immune pathways in sPTB. In contrast, the downregulated DEGs were mainly enriched in glycerolipid biosynthetic process, inflammatory response, immune response-regulating signaling, regulation of phagocytosis, and endocytosis. They were also associated with vesicle/granule-related compartments and molecular functions, such as carbohydrate binding, phosphatase activity, leukocyte migration, and regulation of leukocyte chemotaxis, suggesting that possible alterations in immune effector, trafficking, and migratory functions contributed to PTB. Collectively, these findings suggest that maternal blood transcriptomic changes in sPTB may involve enhanced innate inflammatory signaling together with altered immune regulatory and vesicle-associated pathways.

**Figure 3:**
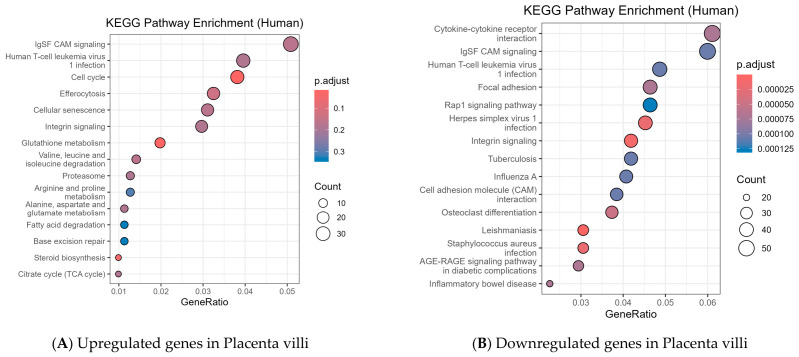

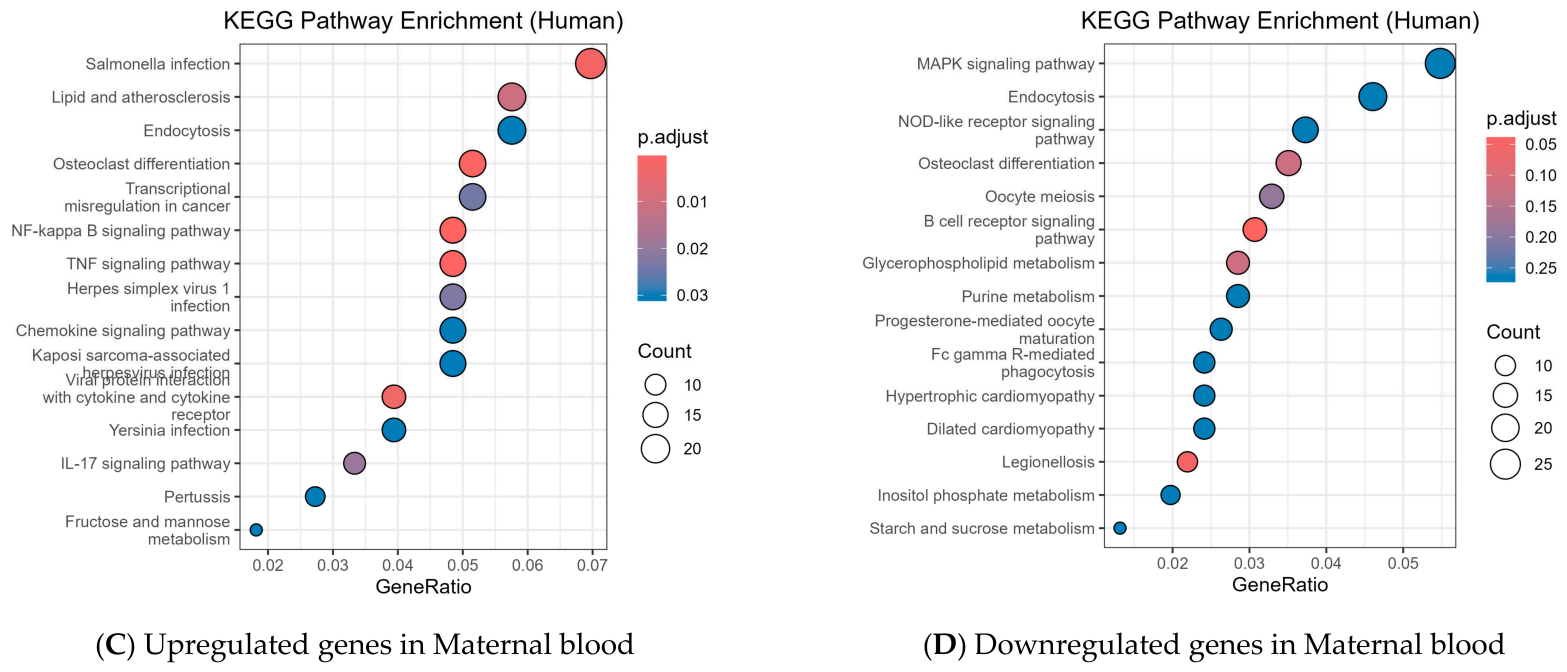
KEGG Pathway enrichment analysis of genes that were identified to be differentially expressed in placental tissues and maternal blood from subjects with spontaneous preterm labor. (**A**) Up-regulated genes in placental villi; (**B**) down-regulated genes in placental villi; (**C**) up-regulated genes in maternal blood; (**D**) down-regulated genes in maternal blood.

KEGG pathway analysis further supported these findings (Figure 3). In placental samples, upregulated genes were enriched in cell cycle, Krebs cycle, fatty acid degradation, steroid biosynthesis, base excision repair, proteasome, and glutathione metabolism pathways. They were consistent with enhanced proliferation, metabolic reprogramming, and stress responses. Downregulated placental genes were mainly enriched in cytokine–cytokine receptor interaction, cell adhesion molecules, focal adhesion, integrin signaling, and Rap1 signaling, indicating impaired immune communication and tissue remodeling at the maternal–fetal interface.

In maternal blood, upregulated DEGs were usually predominantly enriched in pro-inflammatory and infection-related pathways, including interleukin-17 (IL–17), tumor necrosis factor (TNF), nuclear factor kappa B (NF–κB), and chemokine signaling pathways. They were also enriched in viral protein interactions with cytokines and cytokine receptors, such as herpes simplex virus 1 infection, Kaposi sarcoma-associated herpesvirus infection, Salmonella infection, Yersinia infection, and pertussis. This is consistent with the observed heightened systemic innate inflammatory activation in PTB. In contrast, downregulated genes were enriched in Fc gamma receptor (FcγR)-mediated phagocytosis, as well as several metabolism-related pathways, including glycerophospholipid metabolism, purine metabolism, inositol phosphate metabolism, and starch and sucrose metabolism, suggesting attenuation of immune receptor signaling, phagocytic activity, and cellular metabolic programs.

**Figure 4:**
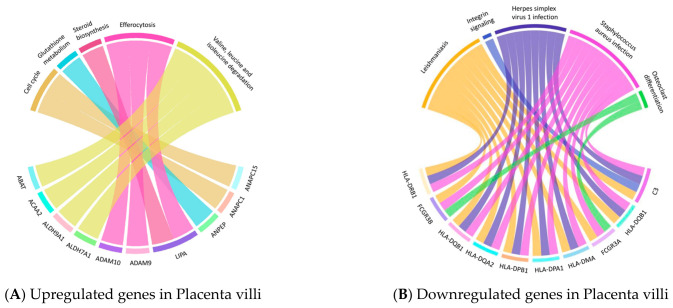

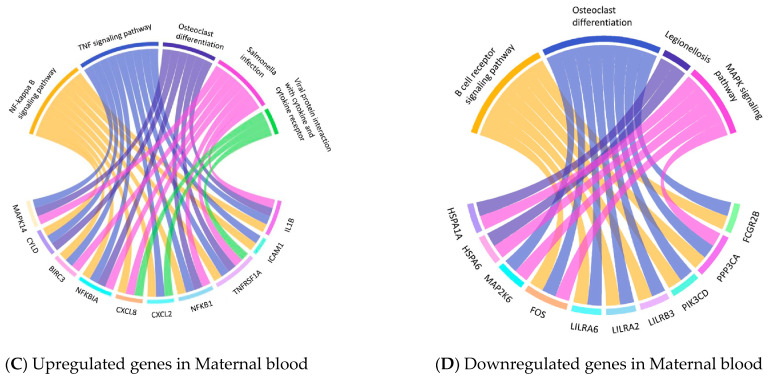
Gene-Pathway Connectivity Network in placental tissues and maternal blood from subjects with spontaneous preterm labor. (**A**) Up-regulated genes in placental villi; (**B**) down-regulated genes in placental villi; (**C**) up-regulated genes in maternal blood; (**D**) down-regulated genes in maternal blood.

KEGG chord plot analysis further highlighted the key genes which were driving the enrichment contributing to the enriched pathway in each tissue type: placenta and maternal blood (Figure 4). In placental villi, upregulated genes, such as *ANAPC1*, *ANAPC15*, *ACAA2*, *LIPA*, *ADAM9*, *ADAM10*, and *ANPEP*, were mainly linked to cell cycle regulation, metabolic pathways, and extracellular remodeling, reinforcing the notion of enhanced proliferative and metabolic activity in preterm placentas. In contrast, placental downregulated genes, including *C3*, *FCGR3A*, *FCGR3B*, *HLA–DMA*, *HLA–DPA1*, *HLA–DPB1*, *HLA–DQA2*, *HLA–DQB1*, and *HLA–DRB1*, were primarily associated with immune regulation, antigen presentation, and cell adhesion-related pathways, indicating impaired local immune communication and maternal–fetal interface remodeling. In maternal blood, upregulated genes, including *IL1B*, *NFKB1*, *NFKBIA*, *CXCL2*, *CXCL8*, *ICAM1*, *TNFRSF1A*, *CYLD*, *BIRC3*, and *MAPK14*, were mainly mapped to the NF–κB and TNF signaling pathways, as well as Salmonella infection, viral protein interactions with cytokines and cytokine receptors, and osteoclast differentiation, indicating activation of inflammatory and innate immune signaling in PTB. In contrast, downregulated genes, including *PPP3CA*, *PIK3CD*, *FCGR2B*, *LILRB3*, *LILRA2*, *LILRA6*, *FOS*, *MAP2K6*, *HSPA1A*, and *HSPA6*, were primarily associated with the B cell receptor signaling pathway, mitogen-activated protein kinase (MAPK) signaling, osteoclast differentiation, and legionellosis-related pathways, suggesting suppression of receptor-mediated immune signaling and downstream stress-response programs. Together, these findings support the presence of systemic immune dysregulation in maternal blood during the PTB process.

**Figure 5:**
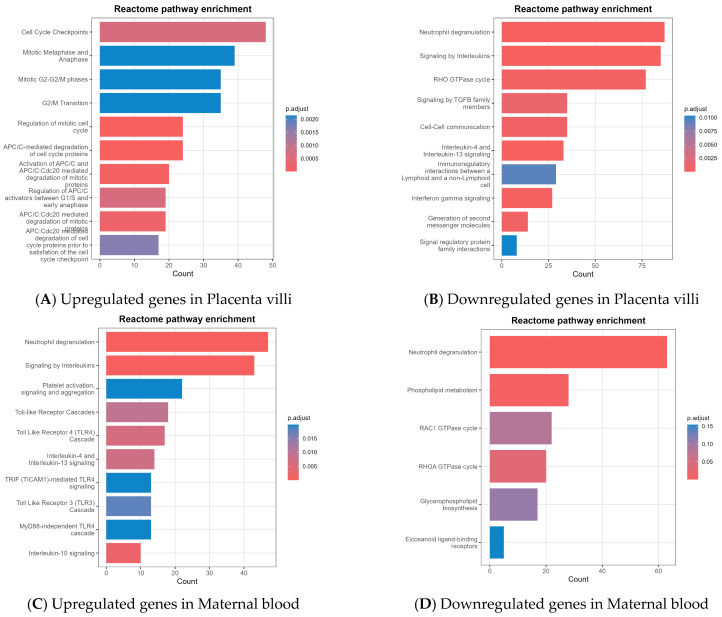
Reactome enrichment analysis of genes that were identified to be differentially expressed in placental tissues and maternal blood from subjects with spontaneous preterm labor. (**A**) Up-regulated genes in placental villi; (**B**) down-regulated genes in placental villi; (**C**) up-regulated genes in maternal blood; (**D**) down-regulated genes in maternal blood.

Reactome pathway analysis showed a similar but more refined functional pattern (Figure 5). In placental villi, upregulated genes were predominantly enriched in pathways related to mitotic cell cycle, cell cycle checkpoints, gap 2/mitosis (G2/M) transition, and anaphase-promoting complex/cyclosome (APC/C)-mediated degradation, further supporting increased cell-cycle activity in preterm placentas. In comparison, placental downregulated genes were enriched in immunoregulatory interactions, interferon-gamma signaling, cell–cell communication, signaling by transforming growth factor beta (TGFB) family members, and RHO guanosine triphosphatase (GTPase)-related pathways, suggesting reduced immune coordination and impaired structural remodeling at the maternal–fetal interface. In maternal blood, upregulated DEGs were mainly enriched in innate immune and inflammatory pathways, including neutrophil degranulation, interleukin signaling, platelet activation, and multiple Toll-like receptor cascades, indicating enhanced immune activation in PTB. In contrast, downregulated DEGs were enriched in phospholipid metabolism, glycerophospholipid biosynthesis, RAC1/RHOA GTPase cycles, and eicosanoid-related pathways, with neutrophil degranulation also represented, suggesting disruption of lipid metabolism, small GTPase signaling, and selected neutrophil effector functions. These findings again support systemic immune reprogramming in maternal blood during PTB.

Overall, integrative GO, KEGG, and Reactome analyses all indicate that PTB is characterized by aberrant placental proliferative/metabolic remodeling and impaired local immune–structural homeostasis, together with systemic maternal immune activation accompanied by dysregulated leukocyte effector programs.

#### 3.4.3. Group C—Pathway Comparison Between PPROM and sPTL

The two studies that specifically compared PPROM and sPTL with intact membranes focused on different gestational tissues. Makieva et al. [56] analyzed cervical biopsies and stratified participants into four groups: PPROM, sPTL with intact membranes, term labor, and term not in labor. Using Illumina HumanHT-12 v4.0 BeadChip (Illumina, San Diego, CA, USA), they demonstrated that the cervical transcriptomic profile of PPROM was clearly distinct from that of sPTL with intact membranes, with 30 upregulated and 9 downregulated in the PPROM group. The PPROM cervix showed increased expression of *PRAM1*, *CEACAM3*, and *FGD3*, together with reduced expression of *NDRG2*. Functional enrichment analyses indicated that the overrepresented programs were predominantly related to immune activation, particularly myeloid- and neutrophil-associated pathways. Importantly, gelatin zymography showed significantly increased MMP–9 activity, whereas MMP–2 was not significantly altered, supporting the concept that PPROM is associated with a specific pattern of cervical remodeling characterized by inflammatory activation, enhanced proteolytic activity, and disruption of ECM homeostasis. These findings suggest that localized cervical stromal remodeling may contribute to focal membrane weakening and rupture at the zone overlying the internal os.

Underhill et al. [57] examined fetal membranes from PTBs and directly compared cases in which PPROM preceded labor with controls presenting with sPTL alone. RNA-seq revealed extensive transcriptomic divergence between the two groups, with 1466 DEGs in the amnion and 484 in the chorion. In the amnion, PPROM was characterized by marked downregulation of collagen- and ECM-related pathways, most notably the ECM–receptor interaction pathway, with several significantly enriched pathways driven by COL-family genes, including *COL1A1*, *COL3A1*, *COL4A4*, and *COL4A6*, indicating structural compromise of the membrane scaffold. In parallel, inflammatory pathways were upregulated in the amnion, including cytokine–cytokine receptor interaction, TNF signaling, and C-X-C motif chemokine ligand (CXCL)-related signaling. In the chorion, PPROM was marked by even stronger upregulation of innate–immune and inflammasome-associated pathways, including chemokine signaling, NOD-like receptor signaling, Toll-like receptor signaling, and cytokine–cytokine receptor interaction, together with increased expression of *GBP5*, *CXCL9*, *ALPL*, *S100A8*, *CASP5*, and *MMP25*. These findings support a compartment-specific model in which PPROM fetal membranes exhibit structural failure in the amnion alongside pronounced inflammatory activation in the chorion.

Taking everything into account, these two studies delineate a possible cross-tissue molecular pattern in which PPROM may involve both structural and inflammatory components: (1) a structural axis, marked by amniotic ECM and collagen downregulation alongside enhanced local matrix degradation, and (2) an inflammatory axis, characterized by heightened innate immune sensing, chemotaxis, and myeloid activation in the chorion and cervix. However, given that this interpretation is based on limited transcriptomic studies, the proposed structural–inflammatory framework should be regarded as hypothesis-generating and requires validation in larger, well-designed transcriptomic studies.

## 4. Discussion

**Figure 6:**
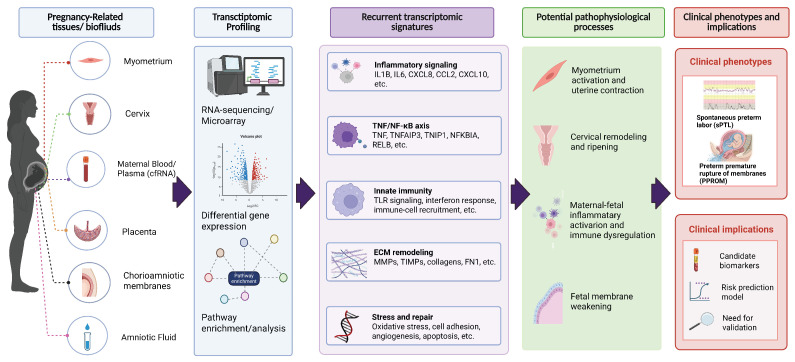
Graphical summary of recurrent transcriptomic signatures across pregnancy-related tissues in sPTL and PPROM. Created in BioRender. Wang, Y. (2026) https://BioRender.com/llr9muu.

This systematic review integrates findings from discovery-oriented transcriptomic studies investigating the biological mechanisms of sPTB, including sPTL and PPROM. Rather than focusing on biomarker validation, these studies were designed to identify novel DEGs and emergent molecular pathways across diverse maternal and gestational tissues. Collectively, they reveal consistent activation of innate immune and inflammatory networks, alongside tissue-specific patterns of ECM remodeling, underscoring both the shared and distinct mechanisms that characterize different PTB phenotypes. To provide an integrated overview, Figure 6 summarizes recurrent transcriptomic signatures across pregnancy-related tissues and their potential relevance to sPTB and PPROM pathophysiology.

### 4.1. Shared Inflammatory Networks Across Gestational Tissues in sPTB

Although placental tissue and maternal blood showed distinct enrichment profiles, both converged on immune–inflammatory dysregulation, particularly cytokine/interleukin signaling and innate immune-related pathways. Placental changes were more strongly associated with impaired local adhesion/remodeling and maternal–fetal interface homeostasis, whereas maternal blood changes primarily reflected systemic inflammatory activation and dysregulated leukocyte effector programs.

At the maternal–fetal interface, innate immune receptor pathways (e.g., pattern-recognition receptors (PRRs) such as Toll-like receptors (TLRs) and NOD-like receptors (NLRs)) can be activated through two non-mutually exclusive routes that are highly relevant to PTB [59,60]. One route is infection-associated inflammation, in which microbial products engage with receptors, such as TLR4, triggering NF–κB-linked inflammatory cascades that have been repeatedly shown to be connected to increased PTB risk [61]. The other route is sterile inflammation, where endogenous danger-associated molecular patterns released from stressed or injured gestational tissues activate PRR/inflammasome-related signaling, producing “infection-like” inflammation even in the absence of detectable pathogens [62]. Enrichment of the cytokine–cytokine receptor interaction pathway suggests the inflammation related to PTB is not confined to the increase in a single factor, but rather is more like a cross-cellular communication network composed of multiple types of cytokines and their receptors being “illuminated” as a whole. Its essence is the “hub layer” where inflammatory signals are maintained and continuously amplified within the tissue [63].

In the reviewed transcriptomic studies, the upregulation of *IL1RL1*, a receptor for the alarmin IL–33 whose functional protein (ST2) is anchored on the cell membrane, further supports the involvement of cytokine-mediated amplification loops that may link tissue stress to sustained inflammatory activation. A 2018 systematic review by Vora et al. [21] identified IL1R1 protein as a characteristic molecule differentially expressed in the peripheral blood of patients with PTB, with this alteration potentially detectable as early as the second trimester. Both *IL1RL1* and *IL1R1* belong to the interleukin-1 receptor family. Although IL1R1 functions as the receptor for the IL–1 axis and IL1RL1/ST2 serves as the receptor for the IL–33 axis, these receptors share the critical co-receptor IL1RAP and largely converge on the canonical MyD88–IRAK–TRAF6–NF–κB/MAPK inflammatory signaling cascade. Consequently, within the same tissue context, signaling through the IL–1 and IL–33 axes often appears highly intertwined, forming overlapping and potentially synergistically amplified inflammatory network nodes [63,64,65]. In our enrichment analysis, the significant enrichment of chemokine signaling suggests that the inflammatory response has progressed beyond upstream activation and entered a stage of active immune cell recruitment. Through binding to their receptors, such as C-X-C chemokine receptor (CXCR) and C-C chemokine receptor (CCR) family members, chemokines establish directional cues for leukocyte migration and represent a central mechanism for immune cell trafficking and tissue localization [66]. This also confirms that during PTB, there can be an increase in infiltration of neutrophils and monocytes/macrophages in the chorionic decidua interface, fetal membranes and uterine-related tissues [67].

### 4.2. Mechanistic Contrasts Between Spontaneous Preterm and Term Labor

A clearer mechanistic understanding emerges when sPTL is compared with physiological TB. Although both processes involve inflammatory activation and uterine transition from quiescence to contractility, transcriptomic analyses consistently indicate that PTB represents a pathological or prematurely activated version of the parturition program, rather than a simple temporal shift in normal term mechanisms [30]. In TB, inflammation is tightly coordinated, locally confined, and embedded within a regulated endocrine–mechanical cascade, including rising fetal signals, progesterone withdrawal pathways, and synchronized myometrial activation [68]. In contrast, the transcriptomic data from sPTL reveal dysregulated or exaggerated inflammatory signaling, premature activation of chemokine and cytokine pathways, and sterile innate immune response that lack the orchestrated hormonal cascade observed in TB [69]. These findings suggest that sPTL is driven by disordered upstream stimuli, such as aberrant immune activation, decidual stress, or myometrial stretching (e.g., secondary to polyhydramnios), rather than engagement of the full physiological parturition program [7].

A parallel distinction is observed when comparing PPROM with term ROM. Term ROM is thought to occur as part of a physiologically timed remodeling process of the amniochorion, involving region-specific ECM turnover, progressive changes in collagen organization and matrix metalloproteinase (MMP) activity, and focal weakening of the membranes in the cervical zone of altered morphology in late gestation [70,71]. In contrast, the transcriptomic studies that were reviewed here, together with broader molecular evidence, suggest that PPROM may involve pathological alterations in fetal membranes. These include increased expression of MMPs and other proteases, reduced expression of multiple collagen genes and ECM pathways, enhanced oxidative-stress and senescence signatures, and strong activation of innate immune and inflammasome-related pathways [11,57]. This constellation of findings suggests that PPROM is not merely “early ROM” but rather reflects pathological tissue injury and immune-mediated ECM breakdown, occurring in the absence of the hormonal and biomechanical cues that characterize term ROM. Moreover, the early appearance of these signatures in maternal blood and cervical or fetal membrane tissues indicates that PPROM may represent a chronic, smoldering process of ECM vulnerability superimposed on exaggerated inflammatory activation [72,73].

### 4.3. Distinct and Shared Molecular Pathways Between PPROM and sPTL

In two transcriptomic studies comparing PPROM and sPTL, a common conclusion was reached: immune pathway activation exists in PPROM, but the aspect of “weakening/degradation of the structural barrier (membranes and/or cervix)” plays a more prominent role that was particularly reflected in ECM remodeling and proteolysis. Multiple studies on fetal membranes and cervices demonstrate robust upregulation of MMPs, reduced expression of collagen isoforms, and enrichment of pathways associated with ECM degradation, focal adhesion, and tissue remodeling. These findings are further supported by zymographic assays showing pronounced collagenolytic activity in PPROM, implicating MMP-mediated weakening of the amniochorion as a key mechanism leading to membrane rupture [74,75].

Similar enriched immune-related pathways were detected in both sPTB preceded by PPROM and sPTL with intact membranes, while they were typically more upregulated in PPROM in comparison. Furthermore, PPROM pathways appear to converge toward a clear etiological pattern, and sPTL demonstrates greater heterogeneity, possibly reflecting multiple upstream triggers such as endocrine activation, sterile inflammation, decidual senescence, and mechanical stretch [76]. It is important to highlight that the stronger and earlier appearance of systemic inflammatory signatures in PPROM suggests that it may represent a chronic immune disorder with early gestational origin, whereas sPTL appears more closely related to late-gestational functional activation. This distinction reinforces the necessity of separating PPROM and sPTL phenotypes in transcriptomic research and in biomarker development.

Overall, the molecular differences between PPROM and sPTL argue strongly for their separation in transcriptomic studies and biomarker development. Although both phenotypes are distinct from TB and involve premature activation of inflammatory and remodeling pathways, PPROM appears to be more closely linked to structural barrier failure and sustained immune dysregulation, whereas sPTL likely reflects a more heterogeneous and functionally activated state.

### 4.4. Feasibility of Transcriptomics in Predicting sPTB Using Maternal Blood

Collectively, the available evidence [52,53,54,55] suggests that mid-gestation maternal transcriptomic profiles, measured either in plasma RNA/cfRNA or peripheral whole blood, may capture signals associated with subsequent sPTB. However, these findings remain preliminary, phenotype-dependent and not yet ready for clinical translation. Plasma-based studies reported moderate discriminatory performance (AUC ~0.76–0.80) as early as 12–24 weeks’ gestation in specific cohort, with functional annotations implicating ECM remodeling, cervical maturation and early perturbations in regulatory networks related to the transition from myometrial quiescence to activation. In contrast, transcriptomic studies on whole blood generally found weaker and more inconsistent effects. Nevertheless, incorporating longitudinal data modestly improved discrimination and highlighted the upregulation of the immune and inflammatory pathways in sPTB. These findings should be interpreted cautiously given the differences in cohort characteristics, sampling time, assay platforms, and validation strategies.

A recurrent observation across studies is the potential heterogeneity by preterm phenotype. Notably, PPROM demonstrated a more robust and stable immune–inflammatory signature at 16–20 weeks, whereas sPTL with intact membranes exhibited weaker and less consistent changes in second-trimester maternal blood, suggesting differences in underlying mechanism and the optimal sampling window. Overall, while mid-gestational transcriptomic signatures may provide useful exploratory information for risk stratification, all reported models remain preliminary, relying largely on internal validation and limited cohorts. Larger, externally validated studies in diverse populations, with careful phenotype stratification and standardized sampling/assay protocols, are required before these approaches can be considered for clinical implementation.

### 4.5. Strengths and Limitations

To our knowledge, this study is the first to perform a cross-tissue integrative analysis of mRNA transcriptomic data in PTB, providing evidence from multiple biological dimensions to explore its underlying mechanisms. However, the number of eligible studies and sample collection time were limited, particularly after stratification by tissue type, and differences in platforms, analytical methods, and raw data availability mean that tissue-specific findings should be interpreted cautiously and require further validation. Additionally, because some raw data were not deposited in public repositories, this part of the research relied solely on the DEG lists and fold-change thresholds provided by the original authors.

## 5. Conclusions

sPTB appears to be associated with recurrent transcriptomic changes in chemokine/cytokine genes, TNF-related regulators, and ECM-associated pathways, including NF–κB signaling, innate immunity, and ECM remodeling. These signatures may differ in magnitude and pattern between PPROM and sPTL, suggesting potential biological heterogeneity. A key unanswered question is whether these pathway activations arise early enough to be reliably detected in maternal blood during mid-gestation for clinically meaningful early prediction and subsequently effective intervention. Further phenotype-specific, longitudinal, and multi-tissue studies are needed to determine which transcriptomic changes are both mechanistically informative and suitable for early sPTB risk screening.

## Figures and Tables

**Table 2:** Genes with significantly increased RNA levels in placental villi among subjects with spontaneous preterm birth, consistently reported in at least three of the six included studies.

Gene	Chim et al. (2012) [41]	Brockway et al. (2019) [42]	Lien et al. (2021) [43]	Couture et al. (2023) [44]	Akram et al. (2022) [45]	Paquett et al. (2023) [13]
*IL1RL1*	15.50	2.14	7.57			
*NFASC*	3.09	2.64	2.14			
*RRM2*			2.39		2.91	1.41
*VEGFA*	4.80	2.46	3.18			

Notes: Shown in column 2 onwards are fold-change values of RNA levels during PTB.

**Table 3:** Genes with significantly decreased RNA levels in placental villi among subjects with spontaneous preterm birth, consistently reported in at least two of the six included studies.

Gene	Chim et al. (2012) [41]	Brockway et al. (2019) [42]	Lien et al. (2021) [43]	Couture et al. (2023) [44]	Akram et al. (2022) [45]	Paquett et al. (2023) [13]	Gene	Chim et al. (2012) [41]	Brockway et al. (2019) [42]	Lien et al. (2021) [43]	Couture et al. (2023) [44]	Akram et al. (2022) [45]	Paquett et al. (2023) [13]
*ACKR3*			0.61			0.69	*ANO6*					0.38	0.81
*AOAH*					0.31	0.75	*ARF1*	0.28					0.95
*ARRDC3*			0.55			0.68	*BBC3*			0.57			0.80
*BCAR3*	0.28		0.70				*BCL2*	0.30		0.57			
*BCL6*			0.42			0.58	*C8orf58*			0.61			0.67
*CRH*			0.17			0.56	*CRIM1*	0.34		0.68			
*CXCR6*			0.49			0.73	*DDB2*			0.73			0.84
*DENND2D*					0.42	0.79	*DNHD1*			0.45		0.34	
*DSP*			0.71			0.70	*DVL1*			0.75			0.67
*FAT2*			0.33			0.31	*FIGN*			0.69			0.70
*FSCN1*					0.27	0.81	*GABRB1*	0.28			0.20		
*GPER1*			0.50			0.64	*GPR37*			0.62			0.71
*HDAC7*			0.69			0.87	*ICAM3*					0.22	0.70
*INHBA*	0.32		0.31				*ITGB6*			0.40			0.50
*JUP*			0.71			0.84	*KCTD11*			0.65			0.82
*KRT18*					0.49	0.74	*LEP*			0.07			0.15
*LRRC1*	0.29		0.67				*NRP1*	0.29				0.25	
*NUDT3*	0.32					0.82	*PARD6B*			0.75			0.74
*PERP*			0.75			0.75	*PIEZO1*			0.65			0.62
*PIM3*			0.73			0.85	*PLEKHG4B*			0.44		0.41	
*PRX*			0.57			0.70	*RAB25*			0.68			0.78
*RAPGEF1*			0.79			0.84	*REEP4*			0.65			0.77
*RNF24*					0.45	0.76	*RORA*	0.32		0.66			
*SF3A2*			0.69			0.91	*SIRPB1*					0.29	0.53
*SKI*			0.71			0.89	*SLC15A3*					0.27	0.80
*SLC16A13*			0.50			0.76	*SLC44A5*					0.19	0.62
*SMIM5*			0.59			0.68	*SUN2*			0.80			0.89
*THBD*			0.56		0.38		*THSD7A*	0.31		0.56			
*TMEM184A*			0.49			0.68	*TMEM43*	0.27		0.75			
*TNFRSF10C*			0.54			0.67	*TNRC6A*			0.78		0.35	
*TRIM22*			0.55			0.73	*TRIP10*			0.67			0.68
*VWCE*			0.42			0.51	*ZNF395*			0.82			0.85
*ZNF592*			0.82			0.84							

Notes: Shown in column 2 onwards are fold-change values of RNA levels during PTB.

**Table 4:** Genes with significantly changes in RNA levels in maternal whole blood among subjects with spontaneous preterm birth, consistently reported in at least two of the six included studies.

Gene	Group B1			Group B2			
	Heng et al. (2014) [49]	Paquette et al. (2018) [50]	Yoo et al. (2021) [51]	Heng et al. (2016) [52]	Weiner et al. (2021) [53]	Gupta et al. (2022) [54]	Camunas et al. (2022) [55]
Upregulated genes							
*ATN1*			1.83		3.20		
*CAPN13*					3.30		2.40
*CST7*	1.36	8.11					
*CYSTM1*	1.34	2.20					
*EREG*		2.97	3.94				
*FAM20A*		11.96	3.05				
*G0S2*	1.69	3.16					
*GPR84*	1.99	2.81					
*IL1B*	1.31	4.03					
*PER1*		2.30	1.95				
*SERPINB2*	1.51	2.97					
*SOCS3*	1.69	5.94					
*SQLE*			2.14			1.51	
Downregulated genes							
*AATK*			0.38		0.12		
*ABHD3*	0.78		0.49				
*APBB3*			0.56		0.12		
*ARL11*			0.56			0.72	
*CARD8*			0.45		0.06		
*EBAG9*	0.69			0.86			
*GPR141*			0.65			0.63	
*GTF2IP4*			0.49				0.85
*LIN7A*			0.28			0.58	
*LSM10*					0.35	0.78	
*RIPK3*			0.54			0.70	
*S1PR4*			0.36			0.70	

Notes: Shown in column 2 onwards are fold-change values of RNA levels during (Group B1) or before (group B2) PTB.

## Data Availability

No new data were created or analyzed in this study. Data sharing is not applicable to this article.

## Supplementary Materials

The following supporting information can be downloaded at: https://www.mdpi.com/article/10.3390/ijms27136006/s1.

## Abbreviations

The following abbreviations are used in this manuscript:

BP	Biological Process
CC	Cellular Component
cfRNA	Cell-free RNA
CS	Cesarean Section
DEGs	Differentially Expressed Genes
ECM	Extracellular Matrix
ELISA	Enzyme-Linked Immunosorbent Assay
FDR	False Discovery Rate
FC	Fold Change
GO	Gene Ontology
GTPase	Guanosine Triphosphatase
KEGG	Kyoto Encyclopedia of Genes and Genomes
MAPK	Mitogen-Activated Protein Kinase
MF	Molecular Function
MMP	Matrix Metalloproteinase
NF–κB	Nuclear Factor Kappa B
PPROM	Preterm Prelabor Rupture Of Membranes
PTB	Preterm Birth
PRR	Pattern-Recognition Receptors
qPCR	quantitative Polymerase Chain Reaction
RNA-seq	RNA sequence
ROM	Rupture Of Membrane
sPTB	Spontaneous Preterm Birth
sPTL	Spontaneous Preterm Labor
TB	Term Birth
TNF	Tumor Necrosis Factor
