# Supplementary material for: Molecular Mechanism and Pathways of Spontaneous Preterm Birth in Different Gestational Tissues: A Systematic Review of Transcriptome Studies
Source: Int J Mol Sci. 2026 Jul 4;27(13):6006. doi: 10.3390/ijms27136006 (PMC13361238; doi:10.3390/ijms27136006)
Supplement: Supplementary file 1 [file ijms-27-06006-s001.zip › ijms-4333061-supplementary.pdf]

TableS1. Quality assessment framework for transcriptomic studies on spontaneous preterm birth

A. Research Quality Assessment Form

**Source standard**	**Assessment item**	**Scoring criteria**
**STROBE/NOS**	Clear definition of PTB	0 = No/unclear; 1 = Clearly defined (<37 weeks or equivalent)
**STROBE/NOS**	Appropriate control group	0 = No/unclear; 1 = Yes (e.g. term birth)
**STROBE/NOS**	Inclusion/exclusion criteria reported	0 = No; 1 = Yes
**STROBE/NOS**	Baseline characteristics described	0 = No; 1 = Partial; 2 = Comprehensive (age, BMI, parity, infection, etc.)
**STROBE/NOS**	Confounders considered/adjusted	0 = No; 1 = Partial; 2 = Yes
**STROBE/NOS**	Sample size justified	0 = No; 1 = Partial; 2 = Yes
**MIAME/MINSEQE**	Biospecimen source described	0 = No; 1 = Yes
**MIAME/MINSEQE**	Sampling timing reported	0 = No; 1 = Yes
**MIAME/MINSEQE**	Sample processing protocol described	0 = No; 1 = Yes
**MIAME/MINSEQE**	RNA quality assessed (e.g. RIN)	0 = No; 1 = Yes
**MIAME/MINSEQE**	RNA quality acceptable	0 = No/unclear; 1 = Yes
**MIAME/MINSEQE**	Platform identified	0 = No; 1 = Yes
**MIAME/MINSEQE**	Library preparation / experimental protocol reported	0 = No; 1 = Yes
**MIAME/MINSEQE**	Sequencing depth / probe coverage adequate	0 = No/unclear; 1 = Partial; 2 = Yes
**Custom (Transcriptomic)**	Raw data quality control performed	0 = No; 1 = Yes
**Custom (Transcriptomic)**	Low-expression/low-quality features filtered	0 = No; 1 = Yes
**Custom (Transcriptomic)**	Normalization method appropriate	0 = No; 1 = Yes
**Custom (Transcriptomic)**	Batch effect assessed/controlled	0 = No; 1 = Yes
**Custom (Transcriptomic)**	Differential expression method appropriate	0 = No; 1 = Yes
**Custom (Transcriptomic)**	Multiple testing correction applied (e.g. FDR)	0 = No; 1 = Yes
**Custom (Transcriptomic)**	Significance threshold predefined	0 = No; 1 = Yes
**Custom (Transcriptomic)**	Functional enrichment/pathway analysis performed	0 = No; 1 = Yes
**Custom (Transcriptomic)**	Independent validation (qPCR/external dataset)	0 = No; 1 = Partial; 2 = Yes
**MIAME/MINSEQE**	Data availability (e.g. GEO accession)	0 = No; 1 = Yes
**Custom (Reproducibility)**	Code/workflow transparency	0 = No; 1 = Partial; 2 = Yes
**STROBE/NOS**	Study limitations discussed	0 = No; 1 = Yes

High quality： ≥75%(≥24), Moderate quality： 50–74%(16-23), Low quality： <50%(<16)

B. Score of each study

Item	Chim (2012)	Brockway (2019)	Lien (2021)	Couture (2023)	Akram (2022)	Paquett (2023)	Rinaldi (2017)	Pereyra (2019)	Arrowsmith (2020)	Bhatti (2021)	Wikstrom (2022)	Heng (2014)	Paquette (2018)	Yoo (2021)	Heng (2016)	Weiner (2021)	Camunas (2022)	Markieva (2017)	Gupta (2022)	Underhill (2024)
Clear definition of PTB	1	1	1	1	1	1	1	1	1	1	1	1	1	1	1	1	1	1	1	1
Appropriate control group	1	1	1	1	1	1	1	1	1	1	1	1	1	1	1	1	1	1	1	1
Inclusion/exclusion criteria reported	1	1	1	1	1	1	0	1	1	1	1	1	1	1	1	1	1	1	1	1
Baseline characteristics described	2	2	2	2	2	2	2	2	2	2	2	2	2	2	2	0	2	1	0	1
Confounders considered/adjusted	1	1	1	2	1	2	1	2	1	1	1	2	1	0	2	0	1	1	1	1
Sample size justified	0	2	2	0	0	0	0	0	0	0	0	2	0	0	2	0	0	0	2	0
Biospecimen source described	1	1	1	1	1	1	1	1	1	1	1	1	1	1	1	1	1	1	1	1
Sampling timing reported	1	1	0	1	1	1	1	1	1	1	1	1	1	1	1	1	1	1	1	1
Sample processing protocol described	1	1	1	1	1	1	1	1	1	1	1	1	1	1	1	0	1	1	1	1
RNA quality assessed	1	1	1	1	1	1	1	1	1	1	1	1	1	1	1	1	1	1	1	1
RNA quality acceptable	1	1	0	1	1	1	0	1	1	0	0	1	0	1	1	0	0	0	1	1
Platform identified	1	1	1	1	1	1	1	1	1	1	1	1	1	1	1	1	1	1	1	1
Library preparation / experimental protocol reported	1	1	1	1	1	1	1	1	1	1	1	1	1	1	1	1	1	1	1	1
Sequencing depth / probe coverage adequate	2	2	0	0	0	2	2	0	2	2	2	2	0	0	2	2	2	2	2	2
Raw data quality control performed	1	1	0	1	1	1	1	1	1	0	1	1	1	1	1	1	1	1	1	1
Low-expression/low-quality features filtered	1	1	0	0	1	1	1	1	1	0	1	1	1	1	1	0	1	1	0	1
Normalization method appropriate	1	1	0	0	0	1	1	1	1	1	1	1	1	1	1	0	1	0	1	0
Batch effect assessed/controlled	0	1	0	1	1	1	1	1	0	1	0	0	0	0	0	0	0	1	0	0
Differential expression method appropriate	1	1	1	1	1	1	1	1	1	1	1	1	1	1	1	1	1	1	1	1
Multiple testing correction applied	1	1	1	1	1	1	0	1	1	1	1	1	1	0	1	1	1	1	1	1
Significance threshold predefined	1	1	1	1	1	1	1	1	1	1	1	1	1	1	1	1	1	1	1	1
Functional enrichment/pathway analysis performed	1	1	1	1	1	1	1	1	1	1	1	1	1	1	1	1	1	1	1	1
Independent validation	1	1	0	1	1	1	1	1	0	0	0	1	1	1	1	2	1	1	0	1
Data availability	1	1	0	0	1	0	1	1	1	0	0	1	1	1	1	0	1	1	1	1
Code/workflow transparency	1	1	0	1	1	1	1	1	1	1	1	1	1	1	1	0	2	1	1	1
Study limitations discussed	1	1	1	1	1	1	1	1	0	1	0	1	1	1	1	1	1	1	1	1
Total	25	27	17	23	23	26	22	26	24	21	22	28	22	21	27	17	24	22	23	23

Table S2. Genes with significantly increased RNA levels in Placental villi among subjects with spontaneous preterm birth, consistently reported in at least two of the six included studies.

**Gene**	**Chim et al.(2012) [41]**	**Brockway et,al.(2019) [42]**	**Lien et al.(2021) [43]**	**Couture et al. (2023) [44]**	**Akram et al. (2022) [45]**	**Paquett et al. (2023) [13]**	**Gene**	**Chim et al.(2012) [41]**	**Brockway et al.(2019) [42]**	**Lien et al.(2021) [43]**	**Couture et al. (2023) [44]**	**Akram et al.(2022) [45]**	**Paquett et al. (2023) [13]**
ACTB			1.46		9.32		*ADAM9*	3.06			6.73		
ADAMTS4		6.06	4.50				*ADCY1*		4.92	5.28			
ALDH1A2		3.73				2.01	*ALPK2*		2.46	2.89			
ANXA1	7.74		1.67				*AP1S3*			1.88			1.30
ARG2			1.97			1.29	*ART4*		2.00			4.92	
ASB2			16.34			2.35	*BAG2*			1.43		2.25	
CCDC125			1.40			1.16	*CCDC141*		2.83	2.99			
CDCA7			1.46			1.23	*CENPA*					23.62	1.42
CHRDL1		111.43	78.25				*CHST2*		4.00	4.03			
CILP		6.06	10.34				*CRLF1*		2.46	6.36			
CTSW		3.73	5.46				*DEPDC1*			2.14			1.28
DHFR					2.14	1.16	*DHX9*	3.06					1.12
DKK1		13.93	16.22				*DYNC2LI1*			1.45			1.26
DYRK4			1.72		3.16		*DYSF*			1.52			1.31
EBPL			1.66		5.24		*ERLEC1*					7.21	1.09
FHOD3		5.28	3.34				*GGT5*		3.25	3.73			
GLB1L2		7.46	15.56				*GLCE*			1.53			1.18
GREB1		34.30	41.64				*GTF3C6*			1.39			1.21
HAND2		2.64	3.25				*IGFBP1*		194.01	203.66			

IL6ST	2.93		1.82				*KCNB1*		3.48	26.72			
KCND2		6.96	12.55				*KIAA1217*					3.46	1.16
LMO7	3.06					1.19	*LUC7L3*	2.93					1.09
MEDAG		17.15	10.20				*MGST1*		3.48	6.87			
MOV10L1		2.14	4.03				*MPP7*			1.59			1.24
MTHFD1			1.58			1.32	*NDUFA6*			1.36			1.12
NID1	2.95		4.17				*OMD*		4.59	30.06			
OXTR		4.92	4.92				*PAEP*		137.19	78.25			
PLXNA4		2.64	3.58				*POLR2K*			1.31			1.09
PPIG	3.07					1.14	*PRL*		78.79	144.01			
PRUNE2		16.00	25.11				*RAB23*	2.99					1.26
RAMP1		5.28	4.72				*RBP4*		19.70	19.56			
RGS22		2.30	5.35				*RIMKLB*	11.4					1.15
RNF6			1.36			1.12	*RXFP1*		18.38	14.93			
SAE1			1.20			1.23	*SCARA5*		19.70	27.86			
SKP2	3.19		1.44				*SLC26A2*	3.09		1.52			
SLC45A4			1.47			1.52	*SREBF2*			1.41			1.27
SSPN			1.95			1.32	*TMEM132C*		4.00	6.87			
TRPC4		5.28	7.31				*VPS45*			1.22			1.07
WT1		11.31	14.12				*XPA*		2.01				1.22
ZBTB8OS					2.08	1.25							

Notes: Shown in column onwards are fold-change values of RNA levels during PTB.

PRISMA 2020 Checklist

Section and Topic	Item #	Checklist item	Location where item is reported
**TITLE**			
Title	1	Identify the report as a systematic review.	Lines 2-4
**ABSTRACT**			
Abstract	2	See the PRISMA 2020 for Abstracts checklist.	Lines 17-38
**INTRODUCTION**			
Rationale	3	Describe the rationale for the review in the context of existing knowledge.	Lines 55-74
Objectives	4	Provide an explicit statement of the objective(s) or question(s) the review addresses.	Lines 75-81
**METHODS**			
Eligibility criteria	5	Specify the inclusion and exclusion criteria for the review and how studies were grouped for the syntheses.	Lines 125-130, 228-244
Information sources	6	Specify all databases, registers, websites, organisations, reference lists and other sources searched or consulted to identify studies. Specify the date when each source was last searched or consulted.	Lines 86-92
Search strategy	7	Present the full search strategies for all databases, registers and websites, including any filters and limits used.	Lines 83-107
Selection process	8	Specify the methods used to decide whether a study met the inclusion criteria of the review, including how many reviewers screened each record and each report retrieved, whether they worked independently, and if applicable, details of automation tools used in the process.	Lines 108-111 Figure 1
Data collection process	9	Specify the methods used to collect data from reports, including how many reviewers collected data from each report, whether they worked independently, any processes for obtaining or confirming data from study investigators, and if applicable, details of automation tools used in the process.	Lines 117-124
Data items	10a	List and define all outcomes for which data were sought. Specify whether all results that were compatible with each outcome domain in each study were sought (e.g. for all measures, time points, analyses), and if not, the methods used to decide which results to collect.	Lines 122-124
	10b	List and define all other variables for which data were sought (e.g. participant and intervention characteristics, funding sources). Describe any assumptions made about any missing or unclear information.	Lines 118-122
Study risk of bias assessment	11	Specify the methods used to assess risk of bias in the included studies, including details of the tool(s) used, how many reviewers assessed each study and whether they worked independently, and if applicable, details of automation tools used in the process.	Lines 111-117 Table S1
Effect measures	12	Specify for each outcome the effect measure(s) (e.g. risk ratio, mean difference) used in the synthesis or presentation of results.	Lines 140-143 Table 1
Synthesis methods	13a	Describe the processes used to decide which studies were eligible for each synthesis (e.g. tabulating the study intervention characteristics and comparing against the planned groups for each synthesis (item #5)).	Lines 125-130
	13b	Describe any methods required to prepare the data for presentation or synthesis, such as handling of missing summary statistics, or data conversions.	Lines 131-143
	13c	Describe any methods used to tabulate or visually display results of individual studies and syntheses.	Table 1; Figure 1; Figures 2-5
	13d	Describe any methods used to synthesize results and provide a rationale for the choice(s). If meta-analysis was performed, describe the model(s), method(s) to identify the presence and extent of statistical heterogeneity, and software package(s) used.	Lines 133-140 Figures 2-5

PRISMA 2020 Checklist

Section and Topic	Item #	Checklist item	Location where item is reported
	13e	Describe any methods used to explore possible causes of heterogeneity among study results (e.g. subgroup analysis, meta-regression).	Lines 125-130
	13f	Describe any sensitivity analyses conducted to assess robustness of the synthesized results.	Not applicable
Reporting bias assessment	14	Describe any methods used to assess risk of bias due to missing results in a synthesis (arising from reporting biases).	Not formally assessed
Certainty assessment	15	Describe any methods used to assess certainty (or confidence) in the body of evidence for an outcome.	Lines 111-117<br>Table S1
**RESULTS**			
Study selection	16a	Describe the results of the search and selection process, from the number of records identified in the search to the number of studies included in the review, ideally using a flow diagram.	Lines 145-152<br>Figure 1
	16b	Cite studies that might appear to meet the inclusion criteria, but which were excluded, and explain why they were excluded.	Lines 151-162
Study characteristics	17	Cite each included study and present its characteristics.	Lines 163-202<br>Table 1
Risk of bias in studies	18	Present assessments of risk of bias for each included study.	Lines 153-165<br>Table S1
Results of individual studies	19	For all outcomes, present, for each study: (a) summary statistics for each group (where appropriate) and (b) an effect estimate and its precision (e.g. confidence/credible interval), ideally using structured tables or plots.	Table 1
Results of syntheses	20a	For each synthesis, briefly summarise the characteristics and risk of bias among contributing studies.	Lines 163-171<br>Lines 213-227<br>Lines 231-244
	20b	Present results of all statistical syntheses conducted. If meta-analysis was done, present for each the summary estimate and its precision (e.g. confidence/credible interval) and measures of statistical heterogeneity. If comparing groups, describe the direction of the effect.	Not applicable
	20c	Present results of all investigations of possible causes of heterogeneity among study results.	Lines 318-324<br>Lines 332-336
	20d	Present results of all sensitivity analyses conducted to assess the robustness of the synthesized results.	Not applicable
Reporting biases	21	Present assessments of risk of bias due to missing results (arising from reporting biases) for each synthesis assessed.	Not formally assessed
Certainty of evidence	22	Present assessments of certainty (or confidence) in the body of evidence for each outcome assessed.	Not formally assessed
**DISCUSSION**			
Discussion	23a	Provide a general interpretation of the results in the context of other evidence.	Lines 610-620
	23b	Discuss any limitations of the evidence included in the review.	Lines 601-610
	23c	Discuss any limitations of the review processes used.	Lines 601-610
	23d	Discuss implications of the results for practice, policy, and future research.	Lines 610-620
**OTHER INFORMATION**			
Registration and	24a	Provide registration information for the review, including register name and registration number, or state that the review was not registered.	Lines 90-92

PRISMA

PRISMA 2020 Checklist

Section and Topic	Item #	Checklist item	Location where item is reported
protocol	24b	Indicate where the review protocol can be accessed, or state that a protocol was not prepared.	Lines 90-92
	24c	Describe and explain any amendments to information provided at registration or in the protocol.	Not applicable
Support	25	Describe sources of financial or non-financial support for the review, and the role of the funders or sponsors in the review.	Line 630
Competing interests	26	Declare any competing interests of review authors.	Line 626
Availability of data, code and other materials	27	Report which of the following are publicly available and where they can be found: template data collection forms; data extracted from included studies; data used for all analyses; analytic code; any other materials used in the review.	Lines 621-625, 634<br>Table S1

From: Page MJ, McKenzie JE, Bossuyt PM, Boutron I, Hoffmann TC, Mulrow CD, et al. The PRISMA 2020 statement: an updated guideline for reporting systematic reviews. BMJ 2021;372:n71. doi: 10.1136/bmj.n71. This work is licensed under CC BY 4.0. To view a copy of this license, visit https://creativecommons.org/licenses/by/4.0/
